# Computational modelling of stem cell–niche interactions facilitates discovery of strategies to enhance tissue regeneration and counteract ageing

**DOI:** 10.1111/febs.15832

**Published:** 2021-05-14

**Authors:** Ilya Potapov, Laura García‐Prat, Srikanth Ravichandran, Pura Muñoz‐Cánoves, Antonio del Sol

**Affiliations:** ^1^ Computational Biology Group Luxembourg Centre for Systems Biomedicine University of Luxembourg Luxembourg; ^2^ Department of Experimental and Health Sciences CIBER on Neurodegenerative Diseases (CIBERNED) Pompeu Fabra University (UPF) Barcelona Spain; ^3^ Spanish National Center on Cardiovascular Research (CNIC) Madrid Spain; ^4^ Princess Margaret Cancer Centre University Health Network Toronto ON M5G2M9 Canada; ^5^ ICREA Barcelona Spain; ^6^ Bizkaia Technology Park CIC bioGUNE Derio Spain; ^7^ Basque Foundation for Science IKERBASQUE Bilbao Spain

**Keywords:** ageing, computational modelling, limb muscle, stem cells, tissue regeneration

## Abstract

The stem cell niche is a specialized microenvironment for stem cells in an adult tissue. The niche provides cues for the maintenance and regulation of stem cell activities and thus presents a target for potential rejuvenating strategies. García‐Prat *et al*. found that in the heterogeneous population of quiescent stem cells of skeletal muscles, a fraction of cells responsible for regeneration and having genuine ‘stemness’ properties deteriorates only in extremely old age. An essential tool used in this analysis of stem cell–niche interactions is the computational tool, NicheHotSpotter, which proved to be instrumental for identifying niche and cell signalling factors that contribute to the maintenance of the pool of genuine quiescent stem cells. NicheHotSpotter predicts candidate factors by analysing signalling interactome and gene regulatory network data in combination with expression profiles. The effect of the niche environment on stem cells is modelled as a mean field of niche cues that induce sustained activation or inhibition of signalling pathways. In this way, NicheHotSpotter has been successful in delineating novel strategies to enhance stemness, which may rejuvenate skeletal muscle cells at the extreme old age.

## Graphical abstract

The status of the resident stem cells (quiescence or active proliferation/differentiation) depends on signals they receive from the surrounding differentiated cells (niche) of the tissue. It was found that the quiescent stem cells in muscles are heterogeneous and that the genuine quiescent state persists into old age. A computational tool, NicheHotSpotter, was instrumental in predicting niche and cell signalling factors maintaining this population of stem cells with high regenerative potential.

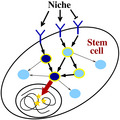

**Abbreviations**
RNA: ribonucleic acid
RNA‐seq: RNA sequencing
TF: transcription factor

## Introduction

The stem cells of an adult organism are located in special microenvironments that enable them to effectively respond to the tissue renewal needs in case of injury and disease. These microenvironments, also known as stem cell niches, provide for the maintenance of the stem cell pool and for the control of the cellular fates during regeneration of an organ or tissue [1, 2]. The stem cell niche interacts with its stem cells through signalling cues that change depending on the regeneration status. Therefore, deciphering niche signals and their effects on the stem cell population is of utmost importance for understanding the maintenance of tissue functions.

Stem cells in a quiescent state play a central role in maintaining the regenerative capacity throughout the lifespan [3, 4, 5]. The quiescent state allows stem cells to survive for long periods and activate in response to demand to renew the surrounding tissue of specialized cells. Reduction in size of the quiescent stem cell pool and disturbance of the cues that maintain the quiescent state both decrease regenerative capacity. These changes intensify with age and disease [6, 7].

## Heterogeneity of muscle stem cells and ageing

In their recent paper ‘FoxO maintains a genuine muscle stem‐cell quiescent state until geriatric age’ [8], García‐Prat *et al*. studied the stem cell heterogeneity in the context of regenerative decline in ageing. The study identifies two quiescent stem cell states in skeletal muscle that emerge in the juvenile age and endure thereafter: a genuine state with stemness properties and a primed state, more committed to myogenic differentiation. These two cellular populations had two distinct transcriptional signatures with CD34 being a biomarker reflecting their differences: high levels of CD34 were characteristic of the genuine state, whereas low levels reflected the primed state. For example, low‐level CD34 cells were shown to have higher levels of *Myod1*, *Actc1* and *Myog*, muscle cell differentiation and maturation‐associated genes. On the other hand, high‐level CD34 cells showed greater clonogenic and self‐renewal capacities, as well as delayed activation kinetics and completion of the first division cycle as compared to the low‐level CD34 cells. Nonetheless, both high‐ and low‐level CD34 cells formed reduced size populations and showed no proliferative traits, suggesting full quiescence.

Next, the authors analysed the dynamism of the stem cell diversity throughout the entire postnatal life of an animal. Thus, in neonatal mice (8 days postbirth) the stem cells were mostly CD34‐negative, which reflects their highly proliferative state. First entrance into quiescence of the stem cells and emergence of the genuine state were observed in juvenile mice (21 days postbirth). The quiescence reaches its height at the young age (2‐ to 4‐month‐old mice), and surprisingly, the genuine‐state cells persist into old (18–22 months) and even extreme old, that is geriatric, mice (28–30 months of age). In conclusion, the genuine stem cell state is preserved into late life; however, it succumbs in geriatric age.

Analysis of the transcriptional signatures revealed that the juvenile cells were closer to the neonatal cells, and together, they were distant from the young cells. Interestingly, neonatal and juvenile stem cells were transcriptionally closer to the primed state, whereas young stem cells were identified closer to the genuine state. Moreover, old (nongeriatric) stem cells are transcriptionally closer to the young genuine quiescent stem cells than to the young primed quiescent stem cells.

Based on this finding, the authors suggest that the decline in the endogenous regenerative potential in old (nongeriatric) muscles is due to the reduction in stem cell number, particularly of primed cells, whereas the more pronounced defect in stem cell function in geriatric muscle is likely due to reduction in both stem cell number and function, particularly affecting the regenerative capacity of genuine quiescent cells. Moreover, the clear transcriptomic difference between the genuine and primed stem cell states is lost in extreme old age.

In order to understand the transcriptional regulation of the primed and genuine states, the authors further studied enrichment of the transcription factor (TF) binding motifs in proximity to the genes defining the core signatures of the two states. The analysis showed enrichment for the Forkhead box O (FoxO) family DNA recognition motif. Additionally, the intracellular FACS assay showed higher abundances of the FoxO family proteins in the genuine‐state cells compared with the primed ones. With the aid of several more assays on mutant mice with deletions of several key FoxO genes, the authors have been able to conclude that the genuine stem cell state is maintained by FoxO TF regulation.

Moreover, the decline in the genuine state at the geriatric age is associated with the decline in FoxO‐related signalling that results in the acquisition of the primed‐state traits. FoxO signalling maintains the expression of the important genuine‐state molecules, whereas FoxO silencing triggers inactivation of the major genuine‐state core genes and expression of genes characteristic of the primed state. FoxO signalling thus maintains the genuine state in adult life and is therefore crucial for maintaining regenerative capacity throughout life, whereas its loss triggers the primed‐state gene expression programme, leading to the depletion of the genuine stem cell pool. In summary, FoxO signalling is crucial to maintain regenerative capacity throughout life.

However, the niche‐mediated signalling cues that potentially regulate the FoxO activity have remained unclear. With the aid of a computational tool, called NicheHotSpotter (also called SigHotSpotter elsewhere [9]), the authors have shown potential niche molecules that maintain the quiescent phenotype of the stem cells by acting on the FoxO TFs. The tool leverages gene expression data to predict the most probable molecules (hot spots) in the signal transduction from the niche via intracellular signalling pathways to the internal phenotypic programmes further defined by the expression of TF genes [9, 10].

## NicheHotSpotter: A tool for predicting stem cell–niche interactions

**Fig. 1:**
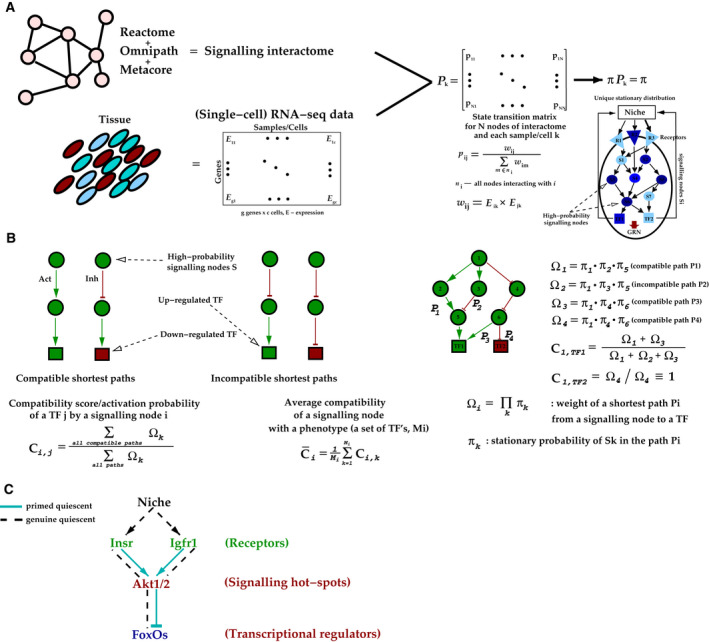
NicheHotSpotter overview and predictions. (A) Calculation of the stationary signalling distribution from the interactome and input gene expression data. TF that have an interactome connection to a signalling node. GRN – gene regulatory network of TFs that do not have interactome connection to signalling nodes. In the stationary probability distribution π, the intensity of blue indicates the stationary probability of the corresponding signalling nodes. (B) Analysis of the compatibility between signalling nodes and target TFs. (C) Schematic summary of the NicheHotSpotter predictions in the muscle stem cells.

NicheHotSpotter integrates gene expression data and the signalling interactome network data combined from multiple sources (such as Omnipath and Reactome) and uses this information to estimate probabilities of network signalling nodes (receptors, kinases, phosphatases, etc.) that mediate sustained niche‐induced signals. Moreover, since signalling cascades are post‐translational events, the method further utilizes the differential TF expression information from two conditions to determine the effects of these signals (Fig. 1A,B).

NicheHotSpotter calculates the probability that a signalling node participates in niche–stem cell signalling from the stationary distribution of a finite discrete time‐homogenous Markov chain model [9, 10]. This model works through a series of layers. NicheHotSpotter starts with a candidate signal released from the niche and tracks the signal to stem cell receptors, with probabilities determined according to the expression level. In this procedure, the probability *p_ij_* of candidate signal transitioning between two interacting nodes *i* and *j* is determined by their expression levels *E_i_* and *E_j_*, respectively. These transition probabilities between all nodes of the signalling interactome are summarized in the state transition matrix *P* (Fig. 1A). The signal is then similarly tracked to the next layer of the signalling nodes of the interactome network, until finally reaching the TF layer. This simulation is repeated multiple times to arrive at a stationary probability distribution for each signalling node (π). At this stage, all nodes in the interactome network are weighed according to the stationary distribution π. Importantly, such a state transition matrix can be constructed from each sample of the gene expression data; hence, every sample (or a cell, in case of the single‐cell data) can be characterized by the stationary distribution π for signalling nodes (Fig. 1A).

The expression level of the signalling nodes alone does not guarantee that signalling events pass through them. In other words, the activity of the signalling molecules is determined by post‐translational modifications (such as phosphorylation), which are the drivers of signal transduction. To address this issue, NicheHotSpotter considers the differential expression of TFs in two conditions being compared. Particularly, at this stage, the most likely signalling hot spots (determined by the stationary distribution π of the Markov chain model; Fig. 1A) are further tested for consistency with the TF signature differences in the two conditions (Fig. 1B). With this approach, NicheHotSpotter scores and classifies highly probable signalling nodes according to their overall compatibility with the TF targets that are ‘closest’ to the hot spots in terms of network proximity (shortest paths). In this regard, hot spots are compatible with their TF targets if their regulatory effect (activation or inhibition) is consistent with the differential gene expression profiles of the targets. Specifically, for each signalling node–TF pair the algorithm calculates the compatibility score by taking into account: i) relative number of shortest paths that are compatible with the TF target and ii) their relative contribution (Ω) determined based on the stationary distribution probabilities of nodes constituting the signalling paths. Calculated this way, the compatibility score reflects activation probability of a target TF by a signalling node. As a result, one can calculate the average compatibility score of any particular node with respect to a set of differentially expressed TFs, which are assumed to constitute the phenotypically relevant differences between the two conditions. NicheHotSpotter thus takes into account not only the expression levels, but also the phenotypic effect of the signalling in question (Fig. 1B).

It should be noted that this approach posits that stem cells are influenced by an average field of all niche factors, including structural, biochemical, and biophysical factors. Furthermore, NicheHotSpotter considers stem cells able to robustly maintain their phenotypic traits by integrating the full complexity of the niche signals into a constant effect of sustained activation and/or inhibition of specific signalling pathways. This mean‐field approach thus avoids the need for detailed characterization of the niche composition or the individual effect of each signalling component on stem cell phenotype.

## NicheHotSpotter predictions define new strategies to enhance tissue regeneration

In their study, García‐Prat *et al*. used NicheHotSpotter tool to predict signalling pathways that maintain the quiescent phenotypes of skeletal muscle stem cells by acting on the FoxO TFs. For the analysis, the authors used RNA‐sequencing (RNA‐seq) expression data from the genuine and primed quiescent stem cell populations. In young mice, NicheHotSpotter predicted that Akt signalling would be inactive in the genuine quiescent stem cell state (the Akt1/2 signalling node was predicted to have the average compatibility score of around 0.31/0.29; see Fig. 1B). Since Akt is activated by phosphorylation [11], consistent with this prediction, the Akt phosphatase PPP2CA, which is known to dephosphorylate Akt in myogenic cells [12], was predicted to be active in these cells. In contrast, the primed quiescent stem cells were shown to have higher amounts of the phosphorylated form of Akt in this study, suggesting signalling activity of Akt in this cellular population (the NicheHotSpotter predictions in this case give the compatibility score of around 0.71/0.72 for Akt1/2). In line with these predictions, the Akt pathway is known to inhibit FoxO transcriptional activity [13]. Moreover, the networks predicted by NicheHotSpotter include several differentially expressed TFs targeted by the FoxO regulators. Based on these analyses, the authors conclude that repressed Akt pathway activity in the genuine quiescent stem cell state activates FoxO TFs and many of their immediate targets in young mice (Fig. 1C).

To confirm these predictions, the authors treated young mice with the Akt inhibitor wortmannin for 2 weeks. This decreased the active form of Akt, while increasing the nuclear content of FoxO molecules. Furthermore, the primed‐state stem cells in the blocker‐treated mice were significantly enriched in molecular signatures of the genuine quiescent stem cell state. Finally, genuine‐ and primed‐state quiescent stem cells from the blocker‐treated young mice both showed increased clonogenic capacity *in vitro* and stem cell engraftment capacity after transplantation *in vivo*. These findings show that Akt inhibition enhances the genuine stem cell state cells, remarkably increasing the regeneration capacity.

NicheHotSpotter has also been instrumental in predicting the putative niche factors that regulate the Akt activity. Among the candidate factors, insulin, insulin growth factor (IGF‐1), and IGF‐1 receptor were shown to activate the signalling network around Akt, suggesting that these niche‐derived factors may inhibit FoxO signalling in the primed‐state quiescent stem cells. Confirming this prediction, skeletal muscle stem cells in transgenic mice overexpressing secreted IGF‐1 in the niche had reduced expression of FoxO molecules, an increased abundance of phosphorylated (active) Akt and altered clonogenic potential (Fig. 1C).

Additionally, genuine‐state stem cells isolated from these mice showed a shift to the expression signatures of the primed‐state cells, when compared to the wild‐type genuine‐state quiescent stem cells. Comparison of data from young and geriatric mice with NicheHotSpotter also predicted elevated activity of insulin/IGF‐1/Akt signalling in the geriatric genuine‐state quiescent stem cells, consistent with the stronger repression of the FoxO TFs in extreme old age (Fig. 1C).

## Conclusion

The results of the García‐Prat *et al*. study demonstrate that NicheHotSpotter can delineate new approaches for increasing the content of genuine‐state quiescent stem cells in skeletal muscle, thus pointing the way to strategies for tissue rejuvenation in extreme old age. NicheHotSpotter is a general tool that can be readily applied to delineate paths towards stem cell rejuvenation in other tissues. In this regard, the tool has already been successfully applied to neural [2, 10], embryonic, haematopoietic and hair‐follicle stem cells, as well as oligodendrocyte progenitor cells [9]. We also note that NicheHotSpotter's potential scope of use can be extended to other tissues as it only requires gene expression data in two conditions. Moreover, as the algorithm does not make any assumptions regarding the nature of data, one can readily use both bulk and single‐cell RNA‐seq data in NicheHotSpotter. However, the interpretation of the results should reflect the data origin. For example, single‐cell data allow studying heterogeneity of cellular populations and have a significant level of dropouts. Hence, one can expect that the high‐probability signalling nodes and their compatibility scores will vary too within a population of cells. This implies additional analysis of such heterogeneity in the context of single‐cell experiments. Finally, in the future this tool can be extended to account for explicit cell–cell interaction events that contribute to the maintenance of stem cell phenotypes in ageing and disease, thus facilitating the design of strategies for tissue regeneration.

## Conflict of interest

The authors declare no conflict of interest.

## Code availability

The source code for NicheHotSpotter is available at: https://gitlab.com/srikanth.ravichandran/signalingfactorscd34positive. The online version of the tool, called SigHotSpotter, along with relevant instructions and test datasets is available at: https://sighotspotter.lcsb.uni.lu/webapp2/.

## Author contributions

IP wrote the manuscript and created figures. LGP wrote the manuscript. SR created figures and wrote the software, including the online version. PMC supervised the work and wrote the manuscript. ADS conceived the idea, supervised the work and wrote the manuscript.
